# 1-Phenyl­isatin

**DOI:** 10.1107/S1600536811027334

**Published:** 2011-07-13

**Authors:** Deepak Shukla, Manju Rajeswaran

**Affiliations:** a Eastman Kodak Company, Kodak Research Laboratories, Rochester, NY 14650-2106, USA

## Abstract

In the title compound, C_14_H_9_NO_2_, the phenyl ring makes a dihedral angle of 50.59 (5)° with the mean plane of the isatin fragment. In the crystal, mol­ecules are linked through weak inter­molecular C—H⋯O hydrogen bonds. The crystal structure also exhibits two slipped π–π inter­actions between the benzene rings of neighbouring mol­ecules [centroid–centroid distance = 3.968 (3) Å, inter­planar distance = 3.484 (3) Å and slippage = 1.899 (3) Å], and between the phenyl rings of neighbouring mol­ecules [centroid–centroid distance = 3.968 (3) Å, inter­planar distance = 3.638 (3) Å and slippage = 1.584 (3) Å].

## Related literature

For the pharmacological properties of isatin derivatives, see: Prakash *et al.* (2010 ▶). For C—C bond lengths in dikotone moieties, see: Rathna & Chandrasekhar, (1991 ▶).
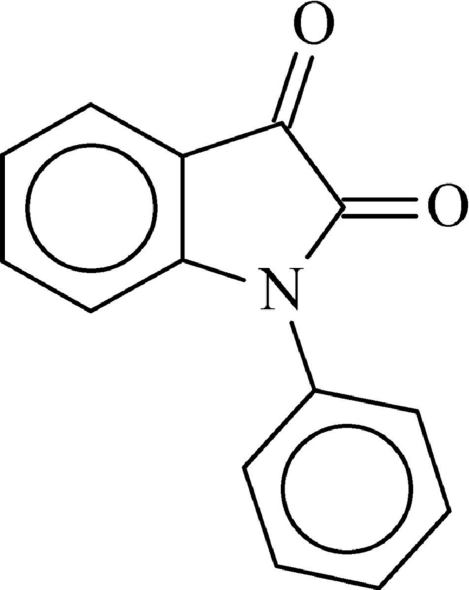


## Experimental

### 

#### Crystal data

C_14_H_9_NO_2_
*M* *_r_* = 223.22
Orthorhombic, 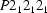

*a* = 3.9677 (1) Å
*b* = 13.3259 (4) Å
*c* = 20.3397 (7) Å
*V* = 1075.42 (6) Å^3^
*Z* = 4
Mo *K*α radiation
μ = 0.09 mm^−1^
*T* = 293 K
0.37 × 0.30 × 0.15 mm

#### Data collection

Nonius KappaCCD diffractometer
7556 measured reflections
1462 independent reflections
1085 reflections with *I* > 2σ(*I*)
*R*_int_ = 0.051

#### Refinement

*R*[*F* ^2^ > 2σ(*F* ^2^)] = 0.038
*wR*(*F* ^2^) = 0.081
*S* = 1.06
1462 reflections
155 parameters
H-atom parameters constrained
Δρ_max_ = 0.16 e Å^−3^
Δρ_min_ = −0.12 e Å^−3^

### 

Data collection: *COLLECT* (Nonius, 2000 ▶); cell refinement: *SCALEPACK* (Otwinowski & Minor, 1997 ▶); data reduction: *DENZO* (Otwinowski & Minor, 1997 ▶) and *SCALEPACK*; program(s) used to solve structure: *SHELXTL* (Sheldrick, 2008 ▶); program(s) used to refine structure: *SHELXTL*; molecular graphics: *SHELXTL*, *Mercury* (Allen *et al.*, 2004 ▶) and *DIAMOND* (Brandenburg, 1998 ▶)’; software used to prepare material for publication: *publCIF* (Westrip, 2010 ▶).

## Supplementary Material

Crystal structure: contains datablock(s) I, global. DOI: 10.1107/S1600536811027334/lx2190sup1.cif

Structure factors: contains datablock(s) I. DOI: 10.1107/S1600536811027334/lx2190Isup2.hkl

Supplementary material file. DOI: 10.1107/S1600536811027334/lx2190Isup3.cml

Additional supplementary materials:  crystallographic information; 3D view; checkCIF report

## Figures and Tables

**Table 1:** Hydrogen-bond geometry (Å, °)

*D*—H⋯*A*	*D*—H	H⋯*A*	*D*⋯*A*	*D*—H⋯*A*
C4—H4⋯O2^i^	0.93	2.58	3.297 (3)	134
C7—H7⋯O1^ii^	0.93	2.52	3.262 (2)	137
C11—H11⋯O2^iii^	0.93	2.58	3.407 (3)	149

Symmetry codes: (i) 
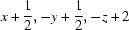
; (ii) 
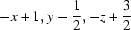
; (iii) 
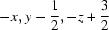
.

**Fig. 1.**
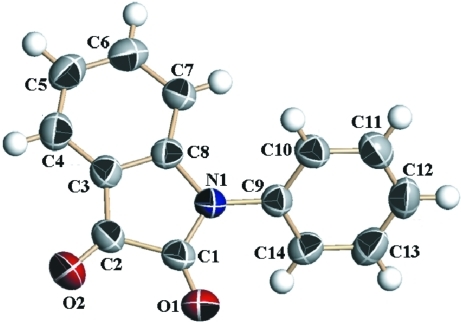
The molecular structure of the title compound with the atom numbering scheme. Displacement ellipsoids are drawn at the 50% probability level. H atoms are presented as a small spheres of arbitrary radius.

**Fig. 2.**
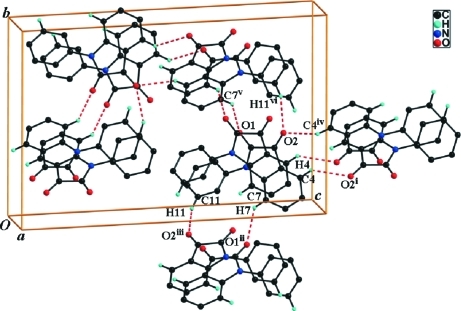
Packing in title compound showing C—H···O interactions. [Symmetry codes: (i) x + 1/2, - y + 1/2, - z + 2; (ii) - x + 1, y - 1/2, - z + 3/2; (iii) - x, y - 1/2, - z + 3/2; (iv) x - 1/2, - y + 1/2, - z + 2; (v) - x + 1, y + 1/2, - z + 3/2; (vi) - x, y + 1/2, - z + 3/2.]

**Fig. 3.**
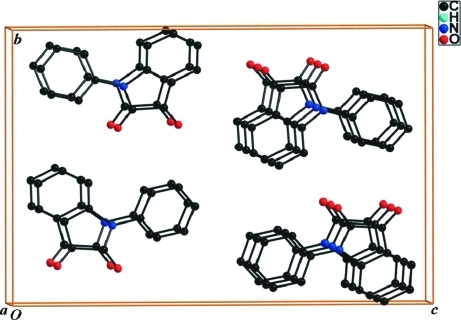
A perspective view of the stacking of title compound in the unit cell viewed down the approximate a axial direction.

**Fig. 4.**
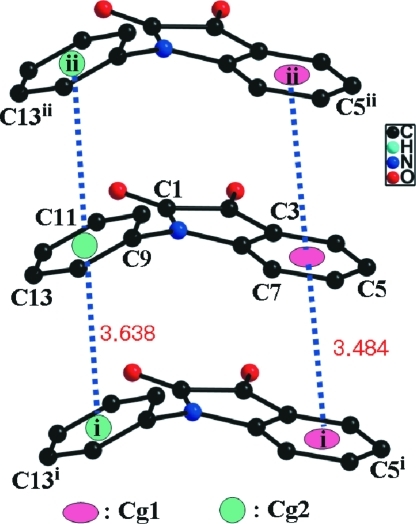
A view of the π···π interactions (dotted lines) in the crystal structure of the title compound. [Symmetry codes: (i) x + 1, y, z; (ii) x - 1, yz.]

## supplementary crystallographic information

### Comment

Isatin is a commercially available indole derivative. Isatin derivatives are well known for their pharmacological properties such as anticonvulsant activity (Prakash *et al.*, 2010). We report herein the crystal structure of the title compound.

In the title compound (Fig. 1), the isatin unit is essentially planar, with a mean deviation of 0.004 (2) Å from the least–squares plane defined by the nine constituent atoms. The phenyl ring makes a dihedral angle of 50.59 (5)° with the mean plane of the isatin fragment. The observed C—C bond length of 1.547 (3) Å in diketo moiety is slightly longer than normal C—C bond length (Rathna & Chandrasekhar, 1991). The crystal packing (Fig. 2) is stabilized by three weak intermolecular C—H···O hydrogen bonds; the first one between a benzene H atom and the O atom of the carbonyl unit (Table 1; C4—H4···O2^i^), the second one between a benzene H atom and the O atom of the carbonyl unit (Table 1; C7—H7···O1^ii^), and the third one between a phenyl H atom and the O atom of the carbonyl unit (Table 1; C11—H11···O2^iii^).

The crystal packing (Fig. 3) is further stabilized by two weak slipped  π···π interactions (Fig. 4); the first one between the benzene rings of neighbouring molecules, with a Cg1···Cg1^i^ distance of 3.968 (3) Å and an interplanar distance of 3.484 (3) Å resulting in a slippage of 1.899 (3) Å (Cg1 is the centroid of the C3–C8 benzene ring), and the second one between the phenyl rings of neighbouring molecules, with a Cg2···Cg2^i^distance of 3.968 (3) Å and an interplanar distance of 3.638 (3) Å resulting in a slippage of 1.584 (3) Å  (Cg2 is the centroid of the C9–C14 phenyl ring)

### Experimental

The title compound, 1–phenylisatin, was purchased from Aldrich Chemical Co.. Single crystals suitable for X—ray diffraction were obtained by sublimation under reduced pressure.

### Refinement

All the Friedel pairs were merged. All H–atoms were positioned geometrically and refined using a riding model with *d*(C—H) = 0.93Å, U_iso_=1.2U_eq_ (C) for aromatic 0.97Å, U_iso_ = 1.2U_eq_ (C) for CH_2_ atoms.

### Crystal data

**Table:**

C_14_H_9_NO_2_	*F*(000) = 464
*M**_r_* = 223.22	*D*_x_ = 1.379 Mg m^−^^3^
Orthorhombic, *P*2_1_2_1_2_1_	Mo *K*α radiation, λ = 0.71073 Å
Hall symbol: P 2ac 2ab	Cell parameters from 4093 reflections
*a* = 3.9677 (1) Å	θ = 1.0–27.5°
*b* = 13.3259 (4) Å	µ = 0.09 mm^−^^1^
*c* = 20.3397 (7) Å	*T* = 293 K
*V* = 1075.42 (6) Å^3^	Rods, orange
*Z* = 4	0.37 × 0.30 × 0.15 mm

### Data collection

**Table:**

Nonius KappaCCD diffractometer	1085 reflections with *I* > 2σ(*I*)
Radiation source: fine-focus sealed tube	*R*_int_ = 0.051
graphite	θ_max_ = 27.4°, θ_min_ = 4.3°
Detector resolution: 9 pixels mm^-1^	*h* = −5→4
φ and ω scans	*k* = −15→17
7556 measured reflections	*l* = −26→25
1462 independent reflections	

### Refinement

**Table:**

Refinement on *F*^2^	Secondary atom site location: difference Fourier map
Least-squares matrix: full	Hydrogen site location: difference Fourier map
*R*[*F*^2^ > 2σ(*F*^2^)] = 0.038	H-atom parameters constrained
*wR*(*F*^2^) = 0.081	*w* = 1/[σ^2^(*F*_o_^2^) + (0.0318*P*)^2^ + 0.1048*P*] where *P* = (*F*_o_^2^ + 2*F*_c_^2^)/3
*S* = 1.06	(Δ/σ)_max_ < 0.001
1462 reflections	Δρ_max_ = 0.16 e Å^−^^3^
155 parameters	Δρ_min_ = −0.12 e Å^−^^3^
0 restraints	Extinction correction: *SHELXTL* (Sheldrick, 2008), Fc^*^=kFc[1+0.001xFc^2^λ^3^/sin(2θ)]^-1/4^
Primary atom site location: structure-invariant direct methods	Extinction coefficient: 0.017 (5)

### Special details

**Table:**

Geometry. All esds (except the esd in the dihedral angle between two l.s. planes) are estimated using the full covariance matrix. The cell esds are taken into account individually in the estimation of esds in distances, angles and torsion angles; correlations between esds in cell parameters are only used when they are defined by crystal symmetry. An approximate (isotropic) treatment of cell esds is used for estimating esds involving l.s. planes.
Refinement. Refinement of F^2^ against ALL reflections. The weighted R-factor wR and goodness of fit S are based on F^2^, conventional R-factors R are based on F, with F set to zero for negative F^2^. The threshold expression of F^2^ > 2sigma(F^2^) is used only for calculating R-factors(gt) etc. and is not relevant to the choice of reflections for refinement. R-factors based on F^2^ are statistically about twice as large as those based on F, and R- factors based on ALL data will be even larger.

### Fractional atomic coordinates and isotropic or equivalent isotropic displacement parameters (Å^2^)

**Table:**

	*x*	*y*	*z*	*U*_iso_*/*U*_eq_	
N1	0.3503 (5)	0.20706 (10)	0.76634 (7)	0.0522 (4)	
O1	0.0729 (4)	0.36020 (10)	0.75768 (7)	0.0685 (4)	
O2	0.0940 (5)	0.34979 (11)	0.90098 (7)	0.0861 (6)	
C1	0.1973 (6)	0.29305 (13)	0.78955 (9)	0.0544 (5)	
C2	0.2117 (6)	0.28662 (15)	0.86534 (9)	0.0589 (6)	
C3	0.3830 (6)	0.19284 (13)	0.87981 (9)	0.0550 (5)	
C4	0.4654 (6)	0.14811 (16)	0.93889 (10)	0.0670 (6)	
H4	0.4099	0.1786	0.9786	0.080*	
C5	0.6316 (7)	0.05730 (16)	0.93787 (11)	0.0714 (7)	
H5	0.6891	0.0258	0.9771	0.086*	
C6	0.7124 (7)	0.01328 (16)	0.87866 (11)	0.0672 (6)	
H6	0.8264	−0.0477	0.8789	0.081*	
C7	0.6296 (6)	0.05666 (13)	0.81841 (10)	0.0572 (5)	
H7	0.6849	0.0258	0.7788	0.069*	
C8	0.4628 (5)	0.14699 (13)	0.82012 (8)	0.0497 (5)	
C9	0.3944 (5)	0.18304 (13)	0.69803 (8)	0.0502 (5)	
C10	0.2965 (6)	0.09053 (14)	0.67389 (10)	0.0591 (6)	
H10	0.2021	0.0428	0.7017	0.071*	
C11	0.3406 (7)	0.06987 (17)	0.60815 (10)	0.0697 (6)	
H11	0.2796	0.0074	0.5916	0.084*	
C12	0.4738 (7)	0.14081 (19)	0.56704 (11)	0.0746 (7)	
H12	0.5012	0.1264	0.5226	0.090*	
C13	0.5670 (7)	0.23267 (17)	0.59071 (10)	0.0706 (7)	
H13	0.6557	0.2806	0.5623	0.085*	
C14	0.5300 (6)	0.25464 (15)	0.65666 (10)	0.0580 (6)	
H14	0.5956	0.3168	0.6730	0.070*	

### Atomic displacement parameters (Å^2^)

**Table:**

	*U*^11^	*U*^22^	*U*^33^	*U*^12^	*U*^13^	*U*^23^
N1	0.0648 (11)	0.0426 (8)	0.0491 (8)	0.0002 (9)	0.0042 (8)	0.0007 (7)
O1	0.0824 (11)	0.0514 (7)	0.0717 (9)	0.0092 (9)	−0.0031 (9)	0.0038 (7)
O2	0.1197 (16)	0.0691 (9)	0.0694 (9)	0.0189 (12)	0.0108 (11)	−0.0142 (8)
C1	0.0609 (13)	0.0437 (9)	0.0585 (11)	−0.0030 (10)	0.0017 (10)	−0.0003 (9)
C2	0.0685 (15)	0.0513 (10)	0.0570 (11)	−0.0023 (12)	0.0054 (11)	−0.0071 (9)
C3	0.0624 (13)	0.0509 (10)	0.0516 (10)	−0.0062 (11)	0.0002 (11)	−0.0008 (9)
C4	0.0776 (17)	0.0688 (12)	0.0547 (12)	−0.0082 (13)	−0.0035 (11)	−0.0005 (10)
C5	0.0798 (18)	0.0711 (13)	0.0632 (14)	−0.0008 (15)	−0.0110 (13)	0.0132 (11)
C6	0.0685 (16)	0.0564 (11)	0.0767 (14)	0.0005 (12)	−0.0096 (13)	0.0088 (11)
C7	0.0619 (13)	0.0499 (10)	0.0598 (12)	0.0004 (11)	0.0024 (12)	−0.0010 (9)
C8	0.0538 (12)	0.0448 (9)	0.0507 (10)	−0.0079 (10)	0.0012 (9)	0.0028 (8)
C9	0.0509 (12)	0.0521 (10)	0.0477 (10)	0.0018 (10)	0.0011 (9)	0.0003 (8)
C10	0.0625 (14)	0.0527 (11)	0.0622 (13)	−0.0027 (11)	0.0032 (12)	−0.0012 (9)
C11	0.0782 (17)	0.0662 (12)	0.0646 (14)	0.0101 (14)	−0.0076 (13)	−0.0139 (11)
C12	0.0856 (19)	0.0907 (16)	0.0475 (11)	0.0272 (16)	−0.0009 (12)	−0.0046 (12)
C13	0.0742 (17)	0.0805 (15)	0.0571 (12)	0.0090 (14)	0.0097 (13)	0.0159 (11)
C14	0.0598 (14)	0.0561 (10)	0.0581 (11)	−0.0023 (11)	0.0022 (11)	0.0069 (9)

### Geometric parameters (Å, °)

**Table:**

N1—C1	1.380 (2)	C6—H6	0.9300
N1—C8	1.427 (2)	C7—C8	1.374 (3)
N1—C9	1.436 (2)	C7—H7	0.9300
O1—C1	1.210 (2)	C9—C14	1.381 (3)
O2—C2	1.205 (2)	C9—C10	1.383 (3)
C1—C2	1.545 (3)	C10—C11	1.376 (3)
C2—C3	1.453 (3)	C10—H10	0.9300
C3—C4	1.381 (3)	C11—C12	1.368 (3)
C3—C8	1.395 (3)	C11—H11	0.9300
C4—C5	1.378 (3)	C12—C13	1.366 (3)
C4—H4	0.9300	C12—H12	0.9300
C5—C6	1.377 (3)	C13—C14	1.381 (3)
C5—H5	0.9300	C13—H13	0.9300
C6—C7	1.394 (3)	C14—H14	0.9300
			
C1—N1—C8	109.94 (15)	C6—C7—H7	121.5
C1—N1—C9	124.71 (15)	C7—C8—C3	120.98 (17)
C8—N1—C9	125.34 (15)	C7—C8—N1	128.50 (16)
O1—C1—N1	127.60 (18)	C3—C8—N1	110.52 (16)
O1—C1—C2	126.19 (18)	C14—C9—C10	120.60 (17)
N1—C1—C2	106.20 (16)	C14—C9—N1	118.86 (16)
O2—C2—C3	131.34 (19)	C10—C9—N1	120.53 (17)
O2—C2—C1	123.17 (19)	C11—C10—C9	119.18 (19)
C3—C2—C1	105.49 (16)	C11—C10—H10	120.4
C4—C3—C8	120.96 (19)	C9—C10—H10	120.4
C4—C3—C2	131.19 (19)	C12—C11—C10	120.3 (2)
C8—C3—C2	107.85 (16)	C12—C11—H11	119.8
C5—C4—C3	118.6 (2)	C10—C11—H11	119.8
C5—C4—H4	120.7	C13—C12—C11	120.5 (2)
C3—C4—H4	120.7	C13—C12—H12	119.7
C6—C5—C4	119.9 (2)	C11—C12—H12	119.7
C6—C5—H5	120.1	C12—C13—C14	120.2 (2)
C4—C5—H5	120.1	C12—C13—H13	119.9
C5—C6—C7	122.5 (2)	C14—C13—H13	119.9
C5—C6—H6	118.8	C13—C14—C9	119.12 (19)
C7—C6—H6	118.8	C13—C14—H14	120.4
C8—C7—C6	117.03 (19)	C9—C14—H14	120.4
C8—C7—H7	121.5		
			
C8—N1—C1—O1	−179.8 (2)	C2—C3—C8—C7	179.5 (2)
C9—N1—C1—O1	1.2 (3)	C4—C3—C8—N1	179.6 (2)
C8—N1—C1—C2	−0.4 (2)	C2—C3—C8—N1	0.0 (2)
C9—N1—C1—C2	−179.38 (19)	C1—N1—C8—C7	−179.2 (2)
O1—C1—C2—O2	0.6 (4)	C9—N1—C8—C7	−0.3 (3)
N1—C1—C2—O2	−178.8 (2)	C1—N1—C8—C3	0.3 (2)
O1—C1—C2—C3	179.8 (2)	C9—N1—C8—C3	179.23 (19)
N1—C1—C2—C3	0.4 (2)	C1—N1—C9—C14	49.4 (3)
O2—C2—C3—C4	−0.6 (4)	C8—N1—C9—C14	−129.4 (2)
C1—C2—C3—C4	−179.8 (2)	C1—N1—C9—C10	−129.4 (2)
O2—C2—C3—C8	178.9 (3)	C8—N1—C9—C10	51.9 (3)
C1—C2—C3—C8	−0.3 (2)	C14—C9—C10—C11	1.0 (3)
C8—C3—C4—C5	0.6 (3)	N1—C9—C10—C11	179.7 (2)
C2—C3—C4—C5	−179.9 (2)	C9—C10—C11—C12	−1.2 (4)
C3—C4—C5—C6	0.1 (4)	C10—C11—C12—C13	0.5 (4)
C4—C5—C6—C7	−0.6 (4)	C11—C12—C13—C14	0.5 (4)
C5—C6—C7—C8	0.3 (4)	C12—C13—C14—C9	−0.7 (4)
C6—C7—C8—C3	0.4 (3)	C10—C9—C14—C13	0.0 (3)
C6—C7—C8—N1	179.8 (2)	N1—C9—C14—C13	−178.8 (2)
C4—C3—C8—C7	−0.9 (3)		

### Hydrogen-bond geometry (Å, °)

**Table:**

*D*—H···*A*	*D*—H	H···*A*	*D*···*A*	*D*—H···*A*
C4—H4···O2^i^	0.93	2.58	3.297 (3)	134.
C7—H7···O1^ii^	0.93	2.52	3.262 (2)	137.
C11—H11···O2^iii^	0.93	2.58	3.407 (3)	149.

Symmetry codes: (i) *x*+1/2, −*y*+1/2, −*z*+2; (ii) −*x*+1, *y*−1/2, −*z*+3/2; (iii) −*x*, *y*−1/2, −*z*+3/2.
